# Fetal growth and risk of childhood asthma and allergic disease

**DOI:** 10.1111/j.1365-2222.2012.03997.x

**Published:** 2012-09-21

**Authors:** S G Tedner, A K Örtqvist, C Almqvist

**Affiliations:** 1 Department of Medical Epidemiology and Biostatistics, Karolinska University Hospital and Karolinska InstitutetStockholm, Sweden; 2 Department of Women's and Children's Health, Astrid Lindgren Children's Hospital, Karolinska University Hospital and Karolinska InstitutetStockholm, Sweden

**Keywords:** allergic diseases, anthropometry, children, epigenetics, fetal growth, fetal programming, infant, low birth weight, twin studies, wheezing

## Abstract

**Introduction:**

Early genetic and environmental factors have been discussed as potential causes for the high prevalence of asthma and allergic disease in the western world, and knowledge on fetal growth and its consequence on future health and disease development is emerging.

**Objective:**

This review article is an attempt to summarize research on fetal growth and risk of asthma and allergic disease. Current knowledge and novel findings will be reviewed and open research questions identified, to give basic scientists, immunologists and clinicians an overview of an emerging research field.

**Methods:**

PubMed-search on pre-defined terms and cross-references.

**Results:**

Several studies have shown a correlation between low birth weight and/or gestational age and asthma and high birth weight and/or gestational age and atopy. The exact mechanism is not yet clear but both environmental and genetic factors seem to contribute to fetal growth. Some of these factors are confounders that can be adjusted for, and twin studies have been very helpful in this context. Suggested mechanisms behind fetal growth are often linked to the feto-maternal circulation, including the development of placenta and umbilical cord. However, the causal link between fetal growth restriction and subsequent asthma and allergic disease remains unexplained. New research regarding the catch-up growth following growth restriction has posited an alternative theory that diseases later on in life result from rapid catch-up growth rather than intrauterine growth restriction *per se*. Several studies have found a correlation between a rapid weight gain after birth and development of asthma or wheezing in childhood.

**Conclusion and clinical relevance:**

Asthma and allergic disease are multifactorial. Several mechanisms seem to influence their development. Additional studies are needed before we fully understand the causal links between fetal growth and development of asthma and allergic diseases.

## Introduction

The prevalence of asthma and allergic disease has increased in many nations around the world 1. The observed marked variation in prevalence between genetically similar populations strongly implies that a substantial proportion is attributable to environmental aspects 2. More than 100 genes are reportedly associated with asthma 3 and a recent genome-wide association study identified six genes with a strong and consistent association, even though the diseases are often influenced by gene–environment interactions 4. Factors related to the hygiene hypothesis 5 have revealed many previously unknown associations and during the past decade the Barker hypothesis, which states that restricted fetal growth may reflect insufficient energy supply for organ development and lead to increased disease susceptibility later in life 1995, has been much discussed.

This area of research is quickly expanding and several mechanisms have been proposed to influence fetal growth. The aim of this article is therefore to summarize current knowledge on fetal growth, and its association to the development of asthma and allergic disease, in order to identify the most urgent research questions and their clinical implications.

## Methods

A PubMed/Medline search was performed with the following MeSH-terms: [children OR child OR childhood] AND [birthweight OR birth weight OR birth length OR head circumference] AND [atopy OR asthma OR atopic OR allergy] AND [fetal programming OR fetal growth] and original articles identified and reviewed. Articles that reported on birth characteristics or fetal growth and asthma/allergic disease in the offspring, particularly those published within the last 10 years, were included and placed in sections related to subject; birth characteristics and asthma/respiratory disease in the offspring, birth characteristics and allergic disease in the offspring; mechanisms that influence fetal growth and their association to asthma or allergic diseases, and catch-up growth or post natal weight gain after fetal growth restriction in association with asthma or respiratory disease. Cross-references were reviewed and additional articles were checked.

## Results

The literature search identified 107 original articles, 29 of which were included in the article. Approximately 350 additional cross-references were reviewed and 24 of them were found relevant. Table 1 displays all these articles. Articles excluded were those only reporting on gestational age/children born prematurely, articles with other main exposures or outcomes, and those published more than 10 years ago. In addition, articles on mechanisms that influence fetal growth and their association to asthma or allergic diseases were assessed and included in the review.

**Table 1:** 29 relevant articles from the original search and 24 cross-references, all reporting on fetal growth/birth characteristics and asthma/respiratory disease/wheezing/atopy/allergic disease in the offspring

Author, ref nr	Study design	Total (% participation)	Age	Results
[Agosti 2003]	Prospective cohort study	83 (82%) and 98 healthy controls	6 years	Lower incidence of allergic symptoms in VLBW children compared to those born full term (31% vs. 52%)
[Anand 2003]	Geographically selected cohort	128 VLBW (32%) and 128 control group	15 years	VLBW had chronic cough, wheezing and asthma and to a higher extent than control group No difference in birth weight ratio and lung function between the groups
[Bardin 2004]	Longitudinal follow up study	37 dyads (SGA + AGA) (80%)	5 years	Prematurely born infants < 28 weeks, study comparing AGA and SGA health status. No major difference in asthma risk first 2 years (24% vs. 30%)
[Benn 2001]	Prospective birth cohort	118 (85%)	5 years	No correlation between large head circumference at birth, thymus size or future development of allergic disease
[Bernsen 2005]	Retrospective	1727 (88%)	6 years	Low birth weight children had a lower risk of atopy, although not significant *P* = 0.07
[Bolte 2004]	Cross-sectional	741 (65%)	5–7 years	Non-significant positive association between birth weight, birth length, gestational age and atopic sensitization in children over 4000 g (OR 1.8; 95% CI 0.8–4.1)
[Brooks 2001]	Cross-sectional	8071 (87%)	3 years	Children with birth weight < 2500 g had a higher risk of asthma 10.9% (OR 1.4; 95% CI 1.1–1.8) than children with a higher birth weight
[Carrington 2006]	Retrospective population-based cohort	256 (47%)	7 years	Reduced odds for wheeze at 7 years in children with head circumference over 36.5 cm at 10-15 days compared to those with head circumference under 35.5 cm (OR 0.12, 95% CI 0.03–0.44, P(trend) = 0.009
[Caudri 2007]	Prospective birth cohort study	3628 (88%)	7 years	A low birth weight was associated with symptoms of respiratory illnesses, OR for every 1000-g decrease in birth weight 1.21 (95% CI 1.09–1.34). The effect of birth weight increased from age 1 to age 5, but then decreased and was no longer significant at age 7.
[Crump 2011]	National cohort	630 090 (97%)	25.5–37 years	Low gestational age associated with a decreased risk of prescribed medications for allergic rhinitis Subjects born w 23–28, adjusted OR 0.70 (95% CI 0.51–0.96) for nasal corticosteroid prescription and 0.45 (95% CI 0.27–0.76) for both nasal corticosteroid and oral antihistamine prescription relative to those born at full term
[Crump 2011]	National cohort study	622 616 (98%)	25.5–35 years	Extremely pre-term children (w 23–27) had more than twice the risk to develop asthma as adults when compared to full-term children; OR 2.4, 95% CI 1.41–4.06)
[Davidson 2010]	Retrospective cohort and follow-up	248 612 birth cohort (98%) and follow-up 4017	10–29 years	Children with a low birth weight (1000–2999 g) had a higher risk of admission to hospital for asthma than children with a birth weight 3000–3999 g, OR 1.2 (95% CI 1.1–1.3)
[Dezateux 2004]	Prospective epidemiological study	234 (22%)	6 weeks	Diminished airway function in children with low birth weight for gestation; a mean reduction of 11 ml in FEV 0.4 (95% CI 4–18; *P* = 0.002), 12 ml in FVC (95% CI 4–19; *P* = 0.004), 28 ml/s in MEF25 (95% CI 7–48; *P* = 0.03)
[Dik 2004]	Retrospective birth cohort	170 960 (92%)	6 years	Increased risk of asthma in children with low birth weight OR 1.08 (95% CI 1.04–1.13) or born pre-term OR 1.28 (95% CI 1.18–1.37), compared to children born full-term and with normal birth weight
[Dombkowski 2008]	Retrospective birth cohort	150 204 (75%)	5–18 years	Pre-term children (< 32 weeks) had a higher risk of asthma 11.7%, regardless of race, compared to full-term (8%) OR 1.51 (95% CI 1.40–1.63)
[Edwards 2003]	Retrospective birth cohort	323 (85%)	45–50 years	Low birth weight predispose for impaired lung function as adults
[Gessner 2007]	Population-based cohort	37349 (68%)	< 5 years and 5–9 years	Pre-term birth but not small for gestational age have an increased risk of asthma
[Gold 1999]	Prospective birth cohort	499	1 year	Significant increased risk of wheezing in children with low birth weight compared to normal weight babies
[Greenough 2004]	Prospective birth cohort	119 (-)	2 years	Pre-term children born small for gestational age (SGA) have different lung function compared to children born with normal weight for gestational age (AGA)
[Hancox 2009]	Population-based cohort	1037 (91%)	32 years	Low birth weight and low weight gain in childhood is associated with modest reduction in lung function in adults
[Hesselmar 2002]	Retrospective case-control study	280 IUGR + 680 controls (63%)	15–25 years	IUGR children develop allergic diseases to the same extent as normal size children
Jakkola 23]	Review and meta-analysis	19 studies		Pre-term delivery results in an increased risk of asthma
Jakkola 14]	Population-based cohort	58841 (98%)	7 years	Low birth weight and pre-term delivery results in increased risk of asthma at age 7. Being small for gestational age is not associated with an increased risk of asthma.
[Jeong 2010]	Hospital based birth cohort	422 (29%)	3 years	Children in tertile with lowest birth weight (OR 3.97; 95% CI 0.94–16.68) and children with highest BMI at check-up (OR 3.68; 95% CI 1.24–10.95) had an increased risk of chronic respiratory illnesses
[Katz 2003]	Retrospective birth cohort Sheffield child development study	10 809 (35%)	11–16 years	A positive correlation between hay fever and: (1) Head circumference (OR 1.2, 95% CI 1.0–1.5) (2) Birth weight (OR 1.2, 95% CI, 1.0–1.4) and (3) Gestational age; children born before 37 weeks had higher risk of hay fever and those with GA > 41 weeks had lower risks, although not significant
[Kawano 2005]	Retrospective case-control study	279 (-)	1 year	Children to allergic mothers tended to have higher gestational age and higher birth weight compared to controls. Allergic children were born with a higher birth weight but shorter gestational age than non-allergic controls (*P* < 0.001)
[Kindlund 2010]	Retrospective twin cohort study with co-twin control analyses	4954 twin pairs (60%)	3–9 years	In twin pairs the twin with lowest birth weight had an increased risk of asthma OR 1.31 (95% CI 1.03–1.65), *P* = 0.027, independent of gestational age
[Laerum 2004]	Retrospective birth cohort study	1683 (53%)	20–44 years	Birth weight showed no relation to adult lung function or symptoms of asthma in adulthood when adjusted for several confounding factors
[Lucas 2004]	Prospective birth cohort	131 (36%)	5–14 weeks	Each SD decrease in birth weight was associated with a 4.4% fall in FEV 0.4s (p = 0.047). When adjusted for FVC, FEV 0.4s fell by 3.2% per SD increase in infant weight gain. This indicate that a slow fetal growth and rapid early infancy weight gain is associated with impaired lung development
[Lundholm 2010]	Register-based twin cohort study with co-twin control analyses	11 020 twins (70%)	9 years 12 years	Positive correlation between birth weight and atopic eczema, OR 1.62 (95% CI 1.27–2.06) for each 500 g increase
[Mallol 2005]	Prospective birth cohort	188 (75%)	1 year	No association between birth weight and recurrent wheezing during first year of life however no child included had a birth weight below 2850g.
Metsälä 20]	Register-based nested case control study	21 038	2–10 years	Low birth weight associated with an increased risk of asthma (OR 1.40, 95% CI 1.20–1.60)
[Nepomnyaschy 2006]	Prospective population based sample study	1803 (37%)	3 years	Children with low birth weight had a higher risk of asthma (34% vs. 18%) than normal weight children
[Nikolajev 2002]	Prospective birth cohort	67 twins (38%)	7–15 years	No correlation between IUGR and bronchial hyperresponsiveness to metacholine when tested at age 7–15 years
[Paul 2010]	Double-blind, randomized, placebo-controlled, parallel-group trial	197 (69%)	2–3 years	Children at risk of asthma with intermittent wheezing were treated with asthma medication or placebo for 2 years. An accelerated weight gain rate lead to more frequent exacerbations but did not affect daily asthma symptoms
[Pekkanen 2001]	Prospective birth cohort	5192 (43%)	31 years	Children born in gestational week > 40 had a higher risk of atopy than children born before 36 weeks of gestation (OR 1.65; 95% CI 1.16–2.34)
[Pike 2010]	Prospective birth cohort	1548 (83%)	3 years	Risk of atopic wheeze increased by 20% per SD decrease in abdominal growth during week 19–34, *P* = 0.046).
[Prabhu 2010]	Longitudinal birth cohort study	1924	5 years	Maternal smoking during pregnancy results in smaller fetal size at birth. Children of mothers that continue to smoke suffers from more episodes of wheezing at the age of 2 years (OR 1.58, *P* = 0.017)
[Raby 2004]	Prospective birth cohort study	454 (91%)	6 years	A positive correlation between low-normal gestational age and asthma at the age of 6 years, OR 4.7 (95% CI 2.1–10.5)
[Rautava 2010]	National cohort study	918 WLBW (73%) and 381 controls	5 years	Very low birth weight children (< 32 weeks or birth weight < 1500 g) had more asthma than controls at check-up
[Rusconi 2007]	Population-based birth cohort	15 609 (69%)	6–7 years	No association between low birth weight < 2500g and wheezing when compared to children with a birth weight of at least 2500 g, OR 1.05 (95% CI 0.81–1.38), 0.96 (95% CI 0.67–1.39), and 0.71 (95% CI 0.49–1.05) for transient early wheezing, persistent wheezing, and late-onset wheezing, respectively
[Sin 2004]	Prospective population-based cohort study	83 595 (87%)	10 years	Children with a high birth weight (above 4500 g) had a higher risk of emergency visits due to asthma than normal weight children, RR 1.16 (95% CI 1.04–1.29)
[Siltanen 2011]	Retrospective Birth cohort	166 (65%) VLBW and 172 (55%) controls	18–27 years	Reduced risk of atopy (positive skin prick test) in children born premature compared to children born full-term OR 0.61(95% CI 0.39–0.93; *P* = 0.023)
[Steffensen 2000]	Population-based study of male conscripts	4795 (99%)	18 years	Higher prevalence of atopic dermatitis in conscripts with low birth weight < 2501 g, OR 3.0 (95% CI 0.8–11.9). Highest incidence of asthma in conscripts with low birth weight < 2500 g
[Tadaki 2009]	Prospective birth cohort study	213 (79%)	1 year	Low birth weight risk factor of wheezing during first year of life OR 1.002 (95% CI 1.000–1.003)
[Taveras 2006]	Prospective birth cohort study	1372 (66%)	2 years	No increased risk of asthma in infants with a birth weight above 4000 g
[Turner 2011]	Longitudinal birth cohort study	1924	10 years	Persistent low growth associated with increased risk of asthma OR 2.8 (95% CI 1.2–6.9) and a mean reduction in FEV1 of 103 ml (95% CI 13–194). Increasing fetal size associated with increased risk of eczema, OR 2.5 (95% CI 1.2–5.3).
[Turner 2010]	Longitudinal birth cohort study	1924	5 years	Smaller fetal size during the first trimester correlated with reduced childhood lung function and increased asthma symptoms, independent of anthropometric measurements at birth and in childhood
[Walter 2009]	Population-based case–control Study	4674 (86%) and 18 445 controls, (85%)	18–27 years	Children with low and moderately low birth weight had a higher risk for hospital admittance due to respiratory problems OR 1.83 (95% CI 1.28–2.62) and OR 1.34 for moderately low birth weight, (95% CI 1.17–1.53)
[Villamor 2009]	Prospective twin cohort study with co-twin control analyses	21 588 twins (66%)	40–72 years	Low birth weight < 2500 g at higher risk of asthma independent of perinatal characteristics. In co-twin control analyses, birth weight of < 2500 g was significantly related to increased risk of asthma among monozygotic twins RR for 2000 g vs. 2500 g OR 1.58 (95% CI 1.06–2.38)
[Yuan 2002]	Retrospective birth cohort study	10 440	12 years	A positive correlation was found between high birth weight and asthma IRR = 1.62, (95% CI 1.02–2.59) per 1000 g increase
[Yuan 2003]	Retrospective birth cohort	9705 (92%)	1 year	An increased risk of anti-asthmatic drugs in children with a high birth weight > 3800 g. (OR 1.23; 95% CI 0.88–1.73)
Örtqvist [35]	Register-based twin cohort study with co-twin control analyses	10 918 twins (69%)	9 and 12 year old twins	Association between low birth weight and increased risk of asthma OR 1.57 (95% CI 1.38–1.79) for each 1000 g decrease in birth weight, with stable estimates in the co-twin analysis

AGA, appropriate for gestational age; CI, confidence interval; FEV, forced expiratory volume; FVC, forced expiratory vital capacity; IRR, incidence rate ratio; IUGR, intrauterine growth restriction; MEF_25_, maximal expired flow at 25% of forced vital capacity; OR, odds ratio; RR, relative risk; SGA, small for gestational age; SD, standard deviation; VLBW, very low birth weight

Several studies have looked at associations between anthropometric measures (birth weight, birth length or head circumference) and asthma or allergic diseases in childhood or adolescence. Most studies have shown a correlation between low birth weight 7–19 and/or gestational age 20,21 and asthma or decreased lung function 10,13,22. Fetal growth and birth weight are not equivalent but a low birth weight can be the result of an impaired fetal growth, or can be due to other factors such as being prematurely born. There is a strong correlation between gestational age and birth weight and, as thoroughly reviewed by Jaakkola et al., children born before 37 weeks of gestation (= preterm) have an increased risk of asthma 2006 so our focus will be on birth anthropometry and fetal growth.

### Birth characteristics and asthma/respiratory disease in the offspring

A prospective population-based study followed children born in large American cities through maternal interviews at 1 and 3 years of age 15. Children with low birth weight had a higher prevalence of asthma than normal weight children (34% vs. 18%). When adjusted for several confounding risk factors, such as socio-economic status, demographics and housing standard, as well as prenatal medical risk factors and behaviours, the risk remained virtually the same (adjusted OR 2.4, *P* < 0.01).

A prospective birth cohort study in Finland found no correlation between gestational age and asthma, but children with a birth weight below 2,500 g had a higher risk of asthma, 12% vs. 8% for those with a higher birth weight 16. The same results have been seen in other studies 7,9,11. When compared to children with normal birth weight, the OR was 1.8 (95% CI 1.3–2.6) for children with a birth weight < 1500 g and 1.3 (95% CI 1.2–1.5) for those weighing 1500–2499 g.

In a prospective birth cohort study from Boston, children born ≥ 36 weeks of gestation, who had one parent with asthma, allergy or hayfever, were followed to the age of 6 years 21. At the first check-up at 1 year, a correlation was found between low birth weight and persistent wheeze. At the age of 6, the correlation was reported between asthma and low-normal gestational age (≤ 38.5 weeks) OR 4.7 (95% CI 2.1–10.5). Furthermore, the study showed that boys of low-normal gestational age were at eightfold greater risk of developing asthma.

Some studies have found that the association between low birth weight/gestational age and asthma/respiratory illnesses seems to weaken with time 8,24. In the PIAMA prospective birth cohort study, children born > 37 weeks of gestation were followed to 7 years of age with annual questionnaires 8. A low birth weight was associated with symptoms of respiratory illnesses, the OR for every 1000-g decrease in birth weight was 1.2 (95% CI 1.1–1.3). However, these results were significant only between the ages 1–5 years, and by age 7, birth weight had no impact. It is possible that the association only exists for a certain period after birth and the effect of fetal growth disappears with age. In line with this a retrospective follow-up study from the United Kingdom collected birth records of 381 men and women, 45–50 years old, and performed a lung function test 24. They found that low birth weight was associated with impaired lung function in terms of forced expiratory volume in 1 s and forced vital capacity, but not with current wheeze.

The associations between birth weight, postnatal growth and adult lung function were assessed in an unselected prospective birth cohort by Hancox et al. 2009. Low birth weight correlated with impaired lung function and lung diffusing capacity. These associations persisted after adjustment for confounding factors including adult weight, exposure to cigarette smoke *in utero* and during childhood, personal smoking, socio-economic status, asthma and gestational age. Children with larger weight gain between birth and age 3 years were more likely to have impaired lung diffusing capacity, indicating that postnatal growth also has an impact on adult lung function.

A prospective birth cohort study assessed the correlation between low birth weight for gestational age and lung function in infancy 10. Children born after 35 weeks of gestation, without respiratory problems at birth or prior to testing, with a birth weight assessed as low or normal for gestational age were examined once at 4–12 weeks of age with measures of weight, crown-heel length, mid-arm, chest circumference and lung function. After controlling for potential confounders, low birth weight children were lighter and shorter than the normal weight group at 6 weeks of age and had lower flow and lung volume parameters.

In the Aberdeen birth cohort study, pregnant women were recruited at a routine ultrasound during the first trimester 22. Fetal crown rump length (CRL) was measured in the first trimester, biparietal diameter in the second trimester and the child completed a spirometric test and a questionnaire at the age of five. The odds of wheezing ever decreased by 4% (95% CI 1–7%; *P* < 0.05) for each millimetre increase in CRL and the odds of having asthma ever decreased by 5% (95% CI 1–9%; *P* < 0.05), indicating that reduced fetal size in the first and second trimester is associated with reduced lung function and increased asthma symptoms at age 5 years. In the recently published 10-year follow-up of the Aberdeen birth cohort, the associations between fetal measurements and asthma remained 18. Compared to fetuses with high CRL at the first trimester and high biparietal diameter in the second trimester (continuous high growth), fetuses with low CRL and biparietal diameter (continuous low growth) had a significant higher risk of asthma and reduced lung function. The study concludes that a continuous high fetal growth rate seems to be a protective factor in the aspect of future asthma development in young adults; OR 2.8 (95% CI 1.2–6.9).

Another group lead by Pike et al. studied fetal growth during different periods in pregnancy in 1548 full-term children 2010. Birth weight and length were measured at birth and growth rates were calculated using the results from the antenatal ultrasound examination. The children were followed up to the age of 3 years. A low growth rate during week 11–19 was associated with an increased risk for non-atopic wheeze (10% per SD decrease) whereas a low growth rate later on in pregnancy (week 19–34) seemed to be associated with atopic wheeze (20% per SD decrease, *P* = 0,046).

Carrington et al. retrospectively collected information from 7-year-old children, registered at two general practitioners clinics in Northampton, United Kingdom, through parental interviews and data extraction from the Personal Child Record Book, a book used by midwives in the United Kingdom at follow-up during infancy 2006. Children born before 36 weeks of gestation were excluded. They found a correlation between a small head circumference at day 10–15 after birth and wheeze at the age of 7, but no association related to head circumference at birth. These results are similar to those of other studies 27,28.

Although several studies have found positive associations between low birth weight and asthma, others have not 24,29,30 and yet some have found the inverse; a correlation between high birth weight and asthma 31,32.

Twin studies provide an excellent opportunity to study the association between fetal growth and asthma, controlling for shared (familial) environmental and genetic factors. Twin siblings share genes (half if dizygotic and all if monozygotic), intrauterine exposures, maternal factors and early environment. In addition, twins often differ in birth weight and body mass index (BMI) over time 33. Since twins have identical GA, any difference in birth weight reflects factors that affected the growth of each individual fetus. If associations seen in a cohort of twins remain in within-pair (co-twin) analyses, then factors specific to each individual must be involved in the underlying causal pathways. Conversely, if the relationships disappear or substantially diminish in within-pair analyses, then factors common to the pair must be involved. Comparison of findings within monozygotic vs. within dizygotic twin pairs (i.e., co-twin control analyses) may provide insights into the role of genetic factors 34.

We have recently shown an increased risk of asthma with a 1000-g decrease in birth weight, OR 1.6 (95% CI 1.4–1.8) 35. A co-twin control analysis was made with 157 monozygotic twins and 289 dizygotic same-sex twin pairs, where a 1000-g decrease in birth weight resulted in an OR of 1.2 (95% CI 0.7–2.1) for the dizygotic same-sex twins and OR 2.4 (95% CI 1.0–6.0) for the monozygotic twins. As genetic and familial factors could be excluded as confounders, these results strengthen the association between childhood asthma and birth weight, indicating that fetal growth restriction might affect early metabolic or physiological mechanisms *in utero*. Another twin study from Sweden on adults 36 and a similar Danish study came to the same conclusion 37.

### Birth characteristics and allergic disease in the offspring

This area is not as explored as for asthma. Most studies have found a positive correlation between gestational age and atopy 16,17,27,38,39 although the correlation between fetal size and eczema is contradictory 18,26.

A cross-sectional study reported a non-significant positive correlation between birth weight, birth length and gestational age and subsequent atopic sensitization 38. The levels of immunoglobulin E (IgE) were elevated and the risk highest in those with a birth weight over 4000 g (OR 1.8; 95% CI 0.8–4.1).

Pekkanen et al. followed children till the age of 31 in a prospective birth cohort study 2001 and found the risk of atopy measured with skin prick test to increase with increasing gestational age. Those born at or above GA 40 weeks had a 65% increased risk of atopy (OR 1.6; 95% CI 1.2–2.3) compared to children born before GA 36 weeks.

Whether or not fetal growth was associated with asthma and atopic dermatitis was examined in a population of male military conscripts in Denmark 17. The prevalence OR of atopic dermatitis among those with a birth weight below 2501 g was 3.0 (95% CI 0.8–11.9) compared with those with a birth weight 3001–3500 g. Those with gestational age below 34 weeks had an adjusted prevalence OR of 0.3 (95% CI 0.0–3.1) suggesting fetal growth retardation rather than preterm delivery as the underlying gestational factor.

Katz et al. studied the presence of hayfever, asthma and eczema in a large cohort of adolescents in Sheffield, United Kingdom, by linking information on birth characteristics to questionnaire data on present symptoms in 11-to-16-year-olds 2003. Hayfever was positively associated with anthropometric measurements such as head circumference OR 1.2 (95% CI 1.0–1.5) and birth weight OR 1.2 (95% CI 1.0–1.4), and also with gestational age; children born before 37 weeks had higher risk of hayfever and those with GA > 41 weeks had lower risks, although not significant. No correlation was found between anthropometric measures and asthma or eczema. Carrington et al. drew the same conclusion regarding eczema 2006 while Turner et al. found a correlation between improved fetal size between first and second trimester and a higher incidence of eczema at the age of 10 years OR 2.5 (95% CI 1.2–5.3) 2011.

In a twin study, we recently found a correlation between birth weight and atopic eczema 40, with an OR of 3.9 (95% CI 1.5–10.0) for a 500-g increase in birth weight, with no significant difference between monozygotic and dizygotic twins (*P* = 0.84). These results, combined with our previous findings of an association between low birth weight and asthma 35, indicate that fetus-specific mechanisms are involved in the aetiology of asthma and atopic eczema.

### Mechanisms that influence fetal growth and their association to asthma or allergic diseases

**Fig. 1:**
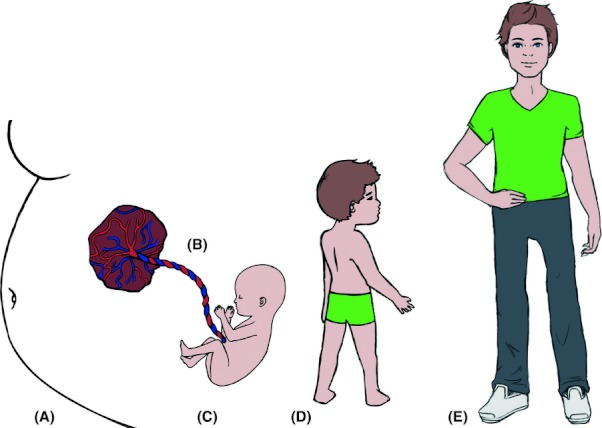
Fetal growth and maternal factors (A), placenta (B), umbilical cord and fetal factors (C) in relation to development of asthma, and allergic disease in childhood (D) and adolescence (E).

Several mechanisms have been suggested to determine fetal growth, most often related to the feto-maternal circulation. Disturbances in the development of the placenta and umbilical cord, on a genetic^41^,^42^, physiological or anatomical level 43–46, inhibit the growth and wellbeing of the fetus. Epigenetic mechanisms are also believed to play a crucial role in the correct development of placenta and fetus by affecting gene expression patterns^47^,^48^ as well as to play a role in the future development of asthma and allergy^47^,^49^. Although much is known about how these factors influence fetal growth, information on the exact mechanisms underlying the association between fetal growth and asthma and allergic disease is sparse. The separate mechanisms are reviewed below, and illustrated in Fig. 1.

#### Maternal factors

Several studies have shown an association between offspring asthma and low socio-economic status^50^,^51^, high maternal BMI^52^,^53^, maternal smoking during pregnancy^14^,^54^, maternal asthma^55^,^56^, maternal intake of paracetamol^57^,^58^, exposure to antibiotics 59, maternal stress during pregnancy 60, maternal intake of vitamin D 61, vitamin E^22^,^62^, folic acid^63^,^64^, and omega-3 polyunsaturated fatty acids^65^,^66^ during pregnancy and mode of delivery 67–69. Twin studies, however, indicate that some of these exposures are rather confounders, since twins share maternal factors but can still differ in size and incidence of asthma/atopic disease 35 (Fig. 1A).

Maternal smoking causes fetal growth retardation 70. Smoking might influence fetal growth to a different extent during different phases of the pregnancy. Prabhu et al. found an exposure-response relationship between cigarettes smoked and fetal femur length in the second and third trimester 2010. Maternal smoking was associated with reduced fetal measurements in the second and third trimester but not in the first trimester. This confirms the researcher's theory that if a pregnant woman stops smoking in the first semester, the fetal size at birth will be normal.

Previous studies have proposed that pregnancy could be associated with a skewing of the immune system from a Th1-type to Th2-type response at the maternal-fetal interface. This skewing may be an essential factor for implantation of the embryo as well as pregnancy itself, by preventing a rejection of the antigenic fetus by the mother's cell-mediated response of Th1 cells 71. Allergic women are characterized by a predisposition to an enhanced Th2 deviation compared to non-allergic women 72. Based on this fact it has been suggested that women with certain atopic phenotypes may have a shorter time to pregnancy 73 and have more successful pregnancy outcomes 74.

#### Placenta

Placental development begins when cytotrophoblast cells attach the embryo to the uterine wall 75–77. The cytotrophoblastic stem cells divide and differentiate into extravillous trophoblastic cells and syncytiotrophoblastic cells. The extravillous trophoblastic cells migrate into the maternal tissue towards the spiral arteries which are remodelled to become stronger and wider, ensuring low resistance. The endovascular trophoblastic cells gather into plugs in the end of the spiral arteries and serve as a filter which prevents maternal blood from flowing into the placenta but permits oxygen supply to the fetus. Oxygenation gradually increases, which stimulates fetal growth and up-regulates several adhesion molecules that facilitate additional trophoblast invasion.

Preeclampsia is a severe complication affecting 2–8% of pregnancies world-wide^78^,^79^ and one of the leading causes of maternal mortality. It is defined as newly arising hypertension (diastolic blood pressure ≥ 90 mm Hg) and proteinuria (≥ 300 mg in 24 h) with an onset at or after 20 weeks of gestation 80. The only treatment is delivery. The exact mechanisms underlying preeclampsia are not yet known but one hypothesis is that disturbed placentation during early pregnancy is followed by release of toxic placental factors that lead to endothelial dysfunction 75–77. In preeclampsia the trophoblastic invasion is shallow and the spiral arteries are not remodelled properly. This leads to an inadequate embryonic blood supply due to non-dilated vessels with high resistance.

Altered levels of angiogenic factors are believed to have an important role in the development of preeclampsia 77. *In vitro* and *in vivo* studies have shown increasing levels of soluble fms-like tyrosine kinase 1 and soluble endoglin and a decrease in the level of vascular endothelial growth factor and placental growth factor. Dysfunctional endothelial cells release vasoactive mediators which perturb haemodynamic function by shifting the balance to vasoconstriction with decreased organ perfusion, oedema and hypertension^77^,^81^. Studies exploring the relationship between preeclampsia and asthma and/or allergy development in the offspring are scarce and have shown conflicting results 69,82,83 (Fig. 1B).

#### Fetal factors

For the fetus preeclampsia can result in intrauterine growth restriction (IUGR) and preterm delivery. IUGR affects about 3–5% of all pregnancies and is believed to occur during the second half of pregnancy 45, although it may be present earlier. Thorsell et al. investigated women with a discrepancy between expected date of delivery calculated from last menstrual period and due date according to ultrasound 2008. If the expected due date calculated from ultrasound was 7 days later than the date calculated from last menstrual period, the baby was twice as likely to be small for gestational age (SGA) at birth, which is in line the hypothesis that small fetal size in early pregnancy may be a result of early growth restriction rather than short gestational duration.

By measuring the uterine artery mean resistance index with Doppler velocimetry during the first trimester, Dugoff et al. found that women with a high mean resistance index had a fivefold higher risk of developing IUGR later in pregnancy (95% CI 1.6–8.7) 2005. This may be a way of identifying patients at risk, who can be followed more closely during the rest of the pregnancy.

One group of children at risk for IUGR is twins and especially monochoriotic twins. Several studies have found that an unequal distribution of the placenta is a major reason for differences in birth weight and selective IUGR in monochoriotic twin pregnancies 43,86,87. This inequality could possibly be an effect of intertwin artery-to-artery anastomoses 88 (Fig. 1C).

In accordance with the finding that allergic disease is associated with shorter waiting time to pregnancy^73^,^74^, higher birth weight and less preterm births^89^,^90^, it has also been suggested that the Th2-like immunity associated with allergic disease could promote a development of Th2 immune responses in the offspring 91.

Research in fetal programming of subsequent disease susceptibility is increasingly focusing on the paradigm that gene regulation beyond the DNA-sequence – epigenetic modulations – could play a key role in the aetiological link between fetal growth and asthma and allergic disease 49,92,93. One type of epigenetic mechanism, where gene expression is determined by a functional inequality of expression between two parental alleles of a gene, is called imprinting. This type of heredity is independent of the Mendelian inheritance, meaning that genes are either expressed only from the allele inherited from the mother, or from the allele inherited from the father. Imprinting of genes has been shown to be an important factor for proper placental development and fetal growth 41,42,48,94. In general, maternally imprinted genes have been shown to restrict fetal growth, whereas paternally imprinted genes enhance fetal growth. The hypothesis behind this distribution of genetic impact is called the genetic conflict hypothesis, suggesting that paternally derived genes influence the fetus to extract more nutrients and energy from the mother for the survival of itself, whereas the maternally derived genes have to balance the energy supply between the current fetus and future fetuses of the same mother. Consequently, the maternally derived genes are more conservative in resource provision 95. Several imprinted genes interact with the growth-controlling systems of insulin-like growth factor and insulin, which in the fetus influence cell proliferation and apoptosis, explaining their role in fetal growth 42 (Fig. 1C).

The transformation of genotype into asthmatic and atopic phenotypes may also be influenced by imprinted genes, such that the predisposition to asthma and allergic disease is present when transferred from the mother, but silenced when transmitted by the father, making maternal history of asthma and allergy more important for the offspring's subsequent morbidity 96. Nevertheless, a recent study by Ferreira et al. showed that the gene for the beta chain of the high affinity IgE receptor was hyper-methylated in all children, independent of their own and their parents' atopic status, showing how difficult it is to assign specific imprinted genes for the inheritance of asthma and allergy 2010, and for the link between fetal growth and asthma and allergic disease.

#### Umbilical cord

Fetal growth impairment is associated not only with disturbances in the placenta, but also with umbilical aberrations. In velamentous cord insertion, the umbilical cord inserts into the fetal membranes, unprotected by Wharton's jelly (WJ), and then travels within the membranes to the placenta instead of inserting into the middle of the placenta as it develops 98. Studies have shown that velamentous cord insertion is associated with impaired fetal growth both in twins^43^,^45^ and in singletons 44. One study has suggested that hormonal and haemodynamic factors that are released under conditions of reduced blood flow could impair fetal growth in a setting of velamentous cord insertion 99 (Fig. 1B and C).

Wharton's Jelly is a gelatinous substance, normally surrounding the umbilical cord, which helps resist external pressure and serves as a physical buffer, regulating the feto-placental circulation 100. A recent study found that if the amount of WJ increased, birth weight also increased 101. The authors suggest that more WJ decreases the probability of cord compression, positively affecting the blood and energy supply to the fetus, thus increasing fetal growth. However, the exact mechanisms behind the relationship between velamentous cord insertion, WJ and impaired fetal growth are incompletely understood (Fig. 1C).

Umbilical cord cytokines are thought to play a key role in regulating the maturation of the fetal immune system, thus conditioning it for postnatal responses against allergens and pathogens. Previous studies have attempted to evaluate the relationship between umbilical cord cytokines, fetal growth 102 and the development of asthma and atopy later in life 103. In one study, higher concentrations of the pro-inflammatory T-helper 1 cytokine interferon-γ (IFN-γ) in umbilical cord blood were found to be associated with a decreased risk of SGA birth 102 (Fig. 1C). The authors suggest that this could indicate a more robust fetal immune response that might arise from a larger and better-nourished fetus, not excluding the possibility of reverse causality, but they also suggest, conversely, that low concentrations of IFN- γ could be a biomarker of placental insufficiency, which in turn could lead to restricted fetal growth. The same study showed that increased concentrations of the pro-inflammatory cytokine tumour necrosis factor α (TNF) were associated with increased risk of preterm delivery, which is not analogous to fetal growth restriction, but can be closely related. The authors here also point out that the results could be an effect of reverse causality, and that the preterm delivery itself leads to higher concentrations of the cytokines. Placental protein levels of IGF-1 and IGFBP-1 are associated with IUGR and also appear to be associated with maternal anthropometry 104,105.

One study of umbilical cord cytokines and the risk of asthma and allergic disease showed that higher concentrations of IFN- γ were protective against asthma in 6-year-old children, but not against atopy, and that higher concentrations of TNF were protective against atopy, but not asthma 103. Assuming that the association between the exposure and outcome is linear, low concentrations of IFN-γ and TNF are associated with a higher risk of asthma and atopy, respectively. These results are in line with our previous findings that fetal growth restriction was associated with an increased risk of asthma 35 but a decreased risk of atopic eczema 40 (Fig. 1D).

#### Thymus

The thymus is a key organ in the human immune system and has long been known to be affected by fetal growth restriction and postnatal malnutrition 106. This has led researchers to investigate if malnutrition *in utero*, measured as restricted fetal growth, can be linked to mismatched development of thymus relative to immune function 107,108. Malnutrition leads to a ‘brain-sparing’ reflex, where blood is redistributed to the brain to maintain oxygen supply, which can result in the birth of a fetus with a disproportionately large head 109. Several studies have shown an association between a large head circumference at birth and increased levels of serum IgE 7,10,11. Godfrey et al. suggest that this could be an effect of a diminished population of Th-1 lymphocytes, caused by impaired thymic maturation during a critical period in fetal development, when other organs are prioritized due to the ‘brain-sparing’ effect 1994 (Fig. 1C). It is possible that immunological mechanisms that can normally be observed during the first year of life, such as a rapid suppression of Th2-responses in non-atopic individuals and a consolidation of Th2-responses in atopic individuals, are not fully developed due to impaired fetal growth 112. On the other hand, in a study by Benn et al., neither the association between a large head circumference and a small thymus, nor the one between small thymus and allergic disease could be confirmed 2001.

#### Dysanapsis

One mechanism that has been much discussed is dysanapsis, the fact that airway size is not necessarily related to lung size 114. Mead et al. found that the airways in adult men were 17% larger than those in adult females 1980. Evidence for this difference in size of airways between men and women has recently been found by Sheel et al., who used computer tomography to measure the respiratory tree 2009. On comparing men and women matched for lung size, women had significantly smaller airway luminal areas. This knowledge of a gender-based difference in the lung composition is not yet known to have a clinical implication for the development of asthma, but may be part of the explanation why asthma is overrepresented in boys but not in adult men 117.

### Catch-up growth and postnatal weight gain after fetal growth restriction in association with asthma or respiratory disease

Catch-up growth in childhood is a strong predictor of non-communicable diseases in adulthood 13,118,119. In a study by Hancox et al., those with the largest weight gain between birth and the age of 3 years were predisposed to an impaired lung diffusing capacity as adults 2009. This is in line with a new theory that it is the rapid catch-up growth following growth restriction that causes the development of diseases later in life, rather than fetal events 120 (Fig. 1D).

Although impaired fetal growth has an independent effect on childhood asthma 35, postnatal weight gain and obesity may also be causally involved in asthma 121. Lucas et al. registered birth weight and crown-heel lengths in 131 normal-term infants 2004, and then measured their lung function at 5–14 weeks of age. In univariate analyses, age-adjusted forced expiratory flow at functional residual capacity was not related to birth weight, but decreased by 11% per SD increase in infant weight gain (*P* < 0.01). The respiratory rate rose by 5% per SD increase in infant weight gain (*P* < 0.01). This is in line with the concept of mismatch, where epigenetic mechanisms have been suggested to play a role 122. As a result of an interaction between genes and the developing milieu during pregnancy, in the processes of developmental plasticity, a fetus is being primed for the predicted postnatal environment. This anticipated environment might be mismatched with the actual postnatal environment that the fetus encounters. When this occurs the ability for an individual to react to environmental encounters might be inadequate and increases the risk of subsequent disease. The degree of mismatch is therefore said to determine the individual's predisposition to chronic disease 123.

To study if infant weight gain patterns after birth were associated with asthma development later in childhood, a randomized prospective study on 2-to-3-year-old children at risk of asthma was performed in the CARE network 124. Birth weight, lung function, growth and symptoms were noted. Weight gain patterns were classified before study enrolment as decelerated, average or accelerated. Participants then received either inhaled corticosteroids or placebo for 2 years. After that period medication was stopped and the children were observed for one more year. Decelerated weight gain patterns were found to be associated with fewer exacerbations (Fig. 1D).

A birth cohort study that investigated children at birth and at the age of three found that the children in the lowest birth weight group had an increased risk of respiratory illness 125. Children who started in the lowest birth weight tertile and who were in the highest weight tertile at age three had a higher risk than those who remained in the middle group. Those with a high BMI at 3 years of age also had an increased risk of respiratory illness.

Most prospective studies on fetal growth follow women from early pregnancy onwards. However, both environmental and genetic factors are known to influence fetal growth and some of these factors might change as the women become pregnant. To address this problem, the Southampton Women's Survey recruited 12 583 women between the ages of 20 and 34 years, measured their characteristics pre-pregnancy, and followed those who got pregnant and also their future children 126. Using this cohort Pike et al. found that adipose weight gain after birth was associated with both atopic and non-atopic wheezing 2010. This supports their theory that different growth rates during pregnancy and after birth have an impact on a child's respiratory health.

Turner et al. studied the relationship between early weight gain, lung function, formula feeding and early asthma 2008. Children introduced to formula milk before the age of 6 months grew more rapidly up to age one than those given breast milk exclusively. Children fed exclusively on breast milk had a lower prevalence of asthma at the age of 3 years than those who received formula milk (OR 0.4; 95% CI 0.2–1.0, *P* < 0.05). However at follow-up at the ages of 6 and 11 years this was no longer significant. It is possible that the weight gain of infants need to be more closely monitored in the future, especially those at risk of developing diseases (Fig. 1E).

## Discussion

In conclusion, a low birth weight has been shown to be associated with asthma 7–9,125. A high birth weight also predisposes for asthma development, particularly when combined with a high BMI later in life^31^,^35^. Growth restriction followed by a rapid catch-up growth has been shown to predispose for impaired lung function and also for asthma 13,22,25,35,121,124,125. Theories differ whether it is the actual fetal growth restriction *in utero* or the rapid catch-up that predisposes for disease development.

No studies investigating the correlation between birth weight and future asthma have been randomized, double-blinded controlled trials (RCT) due to the fact such randomization is ethically and biologically impossible. Most studies are based on data collected retrospectively 7,9,11,19,24,26,29 or prospectively 8,10,13,15,16,21,22. Retrospective study designs have collected data about birth weight in different ways, such as registers or questionnaires 7,9,11,19,24,26,29. There are some inconsistent results between different populations. Reasons might include different outcomes, e g in some studies the outcome is wheeze, in some asthma diagnosis and in others asthma admission 24,29,30,30,31,32. In some studies the follow-up lasts to school age whereas in others it lasts to adulthood. Study population criteria, such as preterm and term children, size of the cohort as well as methods of data collection, potential recall bias and selection, may also affect the results. In addition, a given birth size can be the result of several different antenatal growth trajectories, and thus an infant born weighing 2.5 kg may be constitutively small whereas another weighing 2.5 kg may have been destined to be 3.5 kg and the latter may be at an increased risk for asthma. Today, very few population-based registers for longitudinal measures of growth are available. For reasons of expediency or cost, large-scale studies frequently use self-reported height and weight. However, questions have arisen concerning the validity of these measures, as some participants might over- or underestimate their weight and height 128,129. Data collection through parental questionnaires is another common method within epidemiology. Questionnaire data, however, can suffer from biases (such as misclassification of exposure and recall bias) that decrease the validity of the collected information and may misrepresent the true association between two variables.

Since twins have the same gestational age and often differ in birth weight 33 they are excellent for studies on fetal growth. There might be some concern regarding the generalizability of findings in twins to the general population, because twins are generally more growth restricted *in utero* than singletons 45. For this reason they may have higher rates of asthma later in life. Nevertheless, some previous studies have shown that twins have lower rates of asthma 130,131, whereas other studies have found no difference between twins and singletons 132,133.

When studying disease-discordant twins, these types of confounding factors can be taken into account since twins are generally brought up together and analyses within-twin pairs control for unmeasured environmental and socio-economic factors during childhood.

### Clinical relevance

Fetal growth retardation can be detected ultrasonically and the fetus closely monitored until birth. Children at risk can thus be identified early and followed over time to receive proper medication early if symptoms develop.

### Future studies

To further understand the interplay between genetic and environmental factors in childhood and adolescent asthma and allergic disease, prospective studies should be performed with well characterized subjects. A better understanding of the effect of fetal growth is a key to developing new diagnostic tools as well as prophylactic interventions to reduce the burden of asthma and allergic disease. Twin studies play an important role in the detection of undiscovered genes, but may be even more central in answering the most challenging of all questions: how the environment interacts with genetics in these two outcomes. Future studies may also include women from pre-pregnancy and follow them prospectively during pregnancy and delivery, and follow the offspring in early life, to ascertain measures of fetal growth (anthropometric measures, cord blood and placenta) in relation to subsequent asthma and allergic disease 126,134. This may lead to altered guidelines and improved surveillance of children at risk.
